# Pattern of recurrence of early breast cancer is different according to intrinsic subtype and proliferation index

**DOI:** 10.1186/bcr3559

**Published:** 2013-10-22

**Authors:** Nuria Ribelles, Lidia Perez-Villa, Jose Manuel Jerez, Bella Pajares, Luis Vicioso, Begoña Jimenez, Vanessa de Luque, Leonardo Franco, Elena Gallego, Antonia Marquez, Martina Alvarez, Alfonso Sanchez-Muñoz, Luis Perez-Rivas, Emilio Alba

**Affiliations:** 1 Department of Medical Oncology, Hospital Universitario Virgen de la Victoria, Campus Teatinos s/n, 29010 Málaga, Spain; 2 Department of Pathology, Hospital Universitario Virgen de la Victoria, Campus Teatinos s/n, 29010 Málaga, Spain; 3 Department of Languages and Computer Science, University of Malaga, Campus Teatinos s/n, 29010 Málaga, Spain; 4 Department of Pathology, University of Malaga, Campus Teatinos s/n, 29010 Málaga, Spain

## Abstract

**Introduction:**

Recurrence risk in breast cancer varies throughout the follow-up time. We examined if these changes are related to the level of expression of the proliferation pathway and intrinsic subtypes.

**Methods:**

Expression of estrogen and progesterone receptor, Ki-67, human epidermal growth factor receptor 2 (HER2), epidermal growth factor receptor (EGFR) and cytokeratin 5/6 (CK 5/6) was performed on tissue-microarrays constructed from a large and uniformly managed series of early breast cancer patients (*N* = 1,249). Subtype definitions by four biomarkers were as follows: luminal A (ER + and/or PR+, HER2-, Ki-67 <14), luminal B (ER + and/or PR+, HER2-, Ki-67 ≥14), HER2-enriched (any ER, any PR, HER2+, any Ki-67), triple-negative (ER-, PR-, HER2-, any Ki-67). Subtype definitions by six biomarkers were as follows: luminal A (ER + and/or PR+, HER2-, Ki-67 <14, any CK 5/6, any EGFR), luminal B (ER + and/or PR+, HER2-, Ki-67 ≥14, any CK 5/6, any EGFR), HER2-enriched (ER-, PR-, HER2+, any Ki-67, any CK 5/6, any EGFR), Luminal-HER2 (ER + and/or PR+, HER2+, any Ki-67, any CK 5/6, any EGFR), Basal-like (ER-, PR-, HER2-, any Ki-67, CK5/6+ and/or EGFR+), triple-negative nonbasal (ER-, PR-, HER2-, any Ki-67, CK 5/6-, EGFR-). Each four- or six-marker defined intrinsic subtype was divided in two groups, with Ki-67 <14% or with Ki-67 ≥14%. Recurrence hazard rate function was determined for each intrinsic subtype as a whole and according to Ki-67 value.

**Results:**

Luminal A displayed a slow risk increase, reaching its maximum after three years and then remained steady. Luminal B presented most of its relapses during the first five years. HER2-enriched tumors show a peak of recurrence nearly twenty months post-surgery, with a greater risk in Ki-67 ≥14%. However a second peak occurred at 72 months but the risk magnitude was greater in Ki-67 <14%. Triple negative tumors with low proliferation rate display a smooth risk curve, but with Ki-67 ≥14% show sharp peak at nearly 18 months.

**Conclusions:**

Each intrinsic subtype has a particular pattern of relapses over time which change depending on the level of activation of the proliferation pathway assessed by Ki-67. These findings could have clinical implications both on adjuvant treatment trial design and on the recommendations concerning the surveillance of patients.

## Introduction

The definition of the genomic intrinsic subtypes of breast cancer has been established as the best explanation for the heterogeneous patient outcomes [1-3]. In their original paper, Perou *et al*. [1] found that the genes that differ most between the intrinsic subtypes (luminal A, luminal B, human epidermal growth factor receptor 2 (HER2)-enriched, basal-like and normal-like) were those within the proliferation cluster. In this respect, a meta-analysis of publicly available breast cancer gene expression data, including those from several published prognostic signatures, revealed that more than 70% of the genes associated with patient survival were correlated with the proliferation pathway, whereas 26% were related to estrogen receptor (ER) signaling and 2% to *ERBB2* amplification [4]. The expression of *ESR1-* and *ERBB2*-related genes showed a bimodal distribution; however, this pattern was not observed in proliferative genes. The expression of genes related to proliferation was constitutively high in ER-/HER2- and ER-/HER2+ tumors. However, in ER+/HER2+ tumors, proliferation gene expression occurred along a continuum with a wide range of values from low in relation to that of normal breast tissues to the high values observed in ER-/HER2- or ER-/HER2+ tumors [4]. These data were corroborated by the same group in a later analysis that included more than twice the number of public breast cancer microarrays data sets [5], contributing to the robustness of their findings.

Currently, the applicability of gene expression profiling in clinical practice is extremely limited for technical and economic reasons. Several studies have shown that breast carcinomas can be stratified into subtypes with different prognoses and treatment responses, similarly to those defined by the genomic portraits, using a set of four [6-10] or six [11-15] immunohistochemical markers, including the assessment of a proliferation marker such as Ki-67 in both definitions.

Together with classic prognostic factors, the intrinsic subtype data provide information with which to appraise the total recurrence risk for a given patient. In designing a therapeutic strategy to prevent disease recurrence, however, it is necessary not only to know the total risk of relapse but also likely to ascertain when recurrence is most likely to occur and when the risk becomes minimal. This knowledge could help to establish at what time the administration of adjuvant treatment will be more effective, which should be taken into consideration when developing new adjuvant strategies. There are enough data on the time-varying recurrence risk obtained through analysis using hazard rate functions to support this decision-making. Different authors have described the maximum peak of recurrence risk at 12 to 24 months after surgery [16-22] and the occurrence of a second peak at approximately the fifth year in some cases [17,18,20,22].

In this study, we investigated the importance of the proliferation pathway in the behavior of breast cancer intrinsic subtypes using different statistical approaches. We applied two intrinsic subtype definitions using a set of four or six immunohistochemical markers in a series of early breast cancer patients consecutively treated in a single institution. We also divided every intrinsic subtype according to the level of expression of Ki-67, except in luminal A and luminal B subtypes, because in these cases the Ki-67 expression levels are low and high, respectively, by definition. We hypothesized that a more detailed analysis of the recurrence risk using a hazard rate function methodology would be able to detect differences in such risk over time, depending on the level of expression of the proliferation pathway, even in those subtypes such as HER2-enriched or triple-negative.

## Methods

### Study population

**Figure 1:**
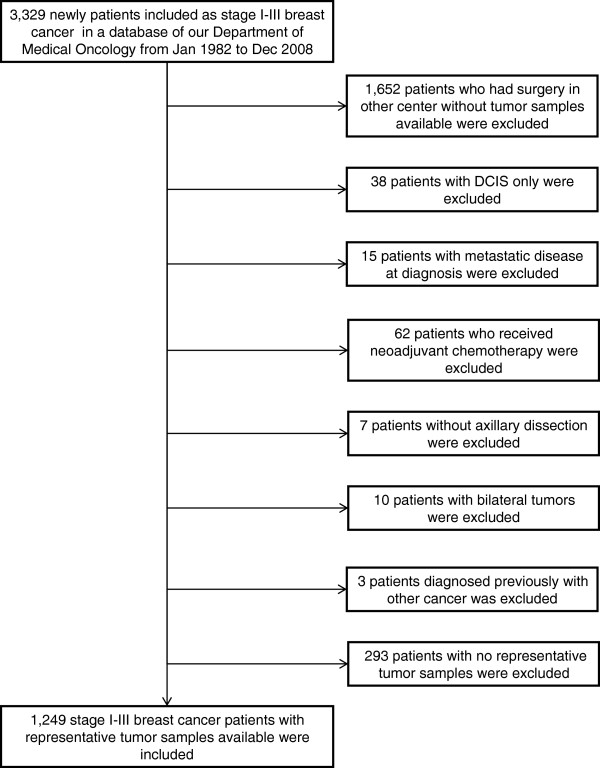
**Flow diagram of the patients through the study.** DCIS, ductal carcinoma *in situ*.

Patients referred to our department were included prospectively in a controlled database. A filtered search was performed to identify stages I to III breast cancer patients enrolled from January 1982 to December 2008. We identified 3,329 patients with stages I to III breast cancer. Nearly 50% (*n* = 1,652) were referred from other centers; consequently, no tumor samples were available. We excluded 135 patients for various reasons, and, among the remaining 1,542 patients, there were no representative tumor samples in 293 cases (19%). Therefore, 1,249 patients were definitively included in the study (Figure 1).

The patients were uniformly treated according to clinical guidelines. Briefly, the chemotherapy regimens used during the 1980s were cyclophosphamide, methotrexate and fluorouracil (CMF); during the 1990s, they were anthracycline-based; and thereafter therapy was anthracycline plus taxane–based. Fifty-seven percent of the patients received adjuvant chemotherapy (CMF, 27.4%; anthracycline-based, 45.9%; taxanes with or without anthracyclines, 25.1%; and unknown 1.6%). Endocrine therapy was administered in 883 patients (luteinizing hormone-releasing hormone analogues plus tamoxifen, 4.5%; tamoxifen, 66.9%; tamoxifen followed by aromatase inhibitor, 19.6%; and aromatase inhibitor 8.9%). Eighteen patients received adjuvant trastuzumab.

Patients underwent follow-up at 6-month intervals during the first 4 years and annually thereafter. Clinicopathological and follow-up information was obtained by chart review.

The study was approved by the Hospital Universitario Virgen de la Victoria Clinical Research Ethics Committee and by the Fondo de Investigaciones Sanitarias from the Ministry of Science and Innovation of Spain under the title “Study of time distribution of recurrence for breast cancer intrinsic subtypes” (PI081797). Informed consent was obtained from patients whose data have been included in this study, except in those cases where the patient had died, in which event the mentioned ethics committees waived the need to obtain informed consent.

### Tissue microarrays and immunohistochemistry

Archival tumor specimens were retrieved, and hematoxylin and eosin sections from each block were reviewed by a pathologist to select representative tumor tissue areas. Tissue microarrays were constructed, and the cases were represented in triplicate with samples from 0.6-mm cores. Immunohistochemical staining was performed for ER (clone SP1; Master Diagnóstica, Granada, Spain), progesterone receptor (PR) (clone Y85; Master Diagnóstica), human epidermal growth factor receptor 2 (HER2) (HercepTest; Dako, Glostrup, Denmark), Ki-67 (clone SP6; Master Diagnóstica), epidermal growth factor receptor (EGFR) (clone EP38Y; Master Diagnóstica) and cytokeratin types 5 and 6 (CK5/6) (clone D5/16B4; Master Diagnóstica). Staining and interpretation of ER, PR, HER2, Ki-67, EGFR and CK5/6 were performed as previously described [23-26]. ER and PR positivity were defined as immunostaining of more than 1% of tumor nuclei. Tumors were considered positive for HER2 if immunostaining was scored as 3+ according to HercepTest criteria. All cases with ambiguous expression of HER2 (HercepTest score 2+) were evaluated by chromogenic *in situ* hybridization (CISH), and an amplification ratio of 2.0 or more was considered as a positive result (HER2 CISH pharmDx; Dako). Ki-67 was visually scored for percentage of tumor cell nuclei with positive immunostaining above the background level. EGFR and CK5/6 stains were considered positive if any (weak or strong) cytoplasmic and/or membranous invasive carcinoma cell staining was observed. Tissue microarrays were scored by two pathologists blinded to the clinicopathological characteristics and outcomes of each patient. The definition of positivity for each of the biomarkers analyzed and the categorization of intrinsic subtypes according to the four or six immunohistochemical variables are summarized in Table 1. The Ki-67 cutoff used was that defined by Cheang *et al*. [12].

**Table 1:** **Immunohistochemical definition of breast cancer intrinsic subtypes according to four or six biomarkers**^**a**^

**Definition and subtype**	**ER**	**PR**	**HER2**	**Ki-67**	**CK5/6**	**EGFR**
Definition of positive result	>1% of tumor nuclei	>1% tumor nuclei	HercepTest score 3+ or 2+ with CISH amplification ratio >2.2	≥14% of tumor nuclei	Any cytoplasmic or membranous staining	Any cytoplasmic or membranous staining
Subtype 4 biomarkers						
Luminal A	ER + or PR+		Negative	Negative	–	–
Luminal B	ER + or PR+		Negative	Positive	–	–
HER2-enriched	Any	Any	Positive	Any	–	–
Triple-negative	Negative	Negative	Negative	Any	–	–
Subtype 6 biomarkers						
Luminal A	ER + or PR+		Negative	Negative	Any	Any
Luminal B	ER + or PR+		Negative	Positive	Any	Any
Luminal HER2	ER + or PR+		Positive	Any	Any	Any
HER2-enriched	Negative	Negative	Positive	Any	Any	Any
Basal-like	Negative	Negative	Negative	Any	CK5/6-positive EGFR-positive	
TNP nonbasal	Negative	Negative	Negative	Any	Negative	Negative

^a^CISH, chromogenic *in situ *hybridization; CK, cytokeratin; EGFR, epidermal growth factor receptor; ER, estrogen receptor; HER2, human epidermal growth factor receptor 2; PR, progesterone receptor; TNP, triple-negative phenotype.

### Statistical analysis methods

The variables analyzed included age, tumor size, histological grade, lymph node status, local therapy, administration of adjuvant chemotherapy, use of adjuvant hormone therapy and intrinsic subtype defined by the four or six biomarkers. The endpoint was breast cancer–free survival (BCFS), defined as the time from surgery until a local, regional or distant recurrence, a second contralateral tumor or death from breast cancer, whichever occurred first. Patients without relapse or who were lost to follow-up were censored at the last follow-up. Patients who died as a result of any cause other than breast cancer were censored at the time of death. All statistical analyses were performed using R version 2.14.0 software [27]. (Last accessed April 29^th^, 2013).

For a more in-depth analysis regarding the importance of the proliferation pathway in the behavior of the intrinsic subtypes of breast cancer, we divided each subtype into two groups: with Ki-67 <14% or with Ki-67 ≥14%. The χ^2^ test was used to compare the distribution of the baseline characteristics among the subgroups. An actuarial survival was performed using the Kaplan-Meier method, and the differences were assessed with logrank, Tarone-Ware and Peto-Peto tests.

The relationships between the various prognostic factors and BCFS were assessed using a Cox proportional hazard regression. Only cases with information for all covariates were included in this analysis (*n* = 1,215). A variable selection was performed using backward and forward stepwise selection processes (the significance level for entry and permanence of a given variable in the model was *P* < 0.05). Among the different candidate Cox models used to analyze the data, the preferred final model was the one with the minimum Akaike information criterion value [28]. The assumption of hazard proportionality for the model was tested, verifying that the prognostic effect of each covariate was proportional throughout the entire follow-up; that is, the effects did not vary over time. A *P*-value less than 0.05 indicated a violation of the proportional hazards assumption. If the assumption of proportional hazards is rejected, it is necessary to study the changes of recurrence risk rates over time. These hazard functions are estimated from right-censored data using kernel-based methods with a global bandwidth selection algorithm and boundary kernel formulations [29]. Afterward it was confirmed that a smoothed hazard function provided a realistic estimation of the underlying hazard function. The results are presented in accordance with the Reporting Recommendations for Tumor Marker prognostic studies [30].

## Results

### Patient cohort

In the final cohort of 1,249 patients, the median follow-up was 73.7 months. There were 344 events (27.5%), of which the first event was distant relapse in 272 cases, locoregional recurrence in 64 cases and contralateral tumors in 8 cases.

Clinicopathological characteristics of the patients with intrinsic subtypes defined by the four biomarkers are shown in Table 2 and those defined by the six biomarkers are given in Table 3. The most frequent intrinsic subtype was luminal A (43.2%), followed by luminal B (30.1%), triple-negative (14.6%) and HER2-enriched (12.1%). Using the six-biomarker definition, there were 6.2% luminal HER2 cases, 5.9% HER2-enriched cases, 10.7% basal-like cases and 3.9% TNP nonbasal cases.

**Table 2:** **Patient characteristics for breast cancer intrinsic subtypes defined by four biomarkers**^**a**^

	**Luminal A and luminal B (*N*** **= 916)**			**HER2-enriched (*N*** **= 151)**			**Triple-negative (*N*** **= 182)**		
**Characteristics**	**Ki-67 <14% (*N*** **= 540)**	**Ki-67 ≥14% (*N*** **= 376)**	***P***^**b**^	**Ki-67 <14% (*N*** **= 42)**^**c**^	**Ki-67 ≥14% (*N*** **= 107)**^**c**^	***P***^**b**^	**Ki-67 <14% (*N*** **= 38)**^**d**^	**Ki-67 ≥14% (*N*** **= 143)**^**d**^	***P***^**b**^
Age (years), *n* (%)									
<40	33 (6.1)	32 (8.5)	NS	4 (9.5)	15 (14.0)	NS	5 (13.2)	22 (15.4)	NS
40 to 55	197 (36.5)	139 (37.1)		14 (33.3)	49 (45.8)		9 (23.7)	58 (40.6)	
>55	310 (57.3)	205 (54.4)		24 (57.1)	43 (40.2)		24 (63.2)	63 (44.1)	
Menopausal status, *n* (%)									
Premenopausal	172 (31.9)	130 (34.7)	NS	15 (35.7)	53 (49.5)	NS	12 (31.6)	59 (41.3)	NS
Postmenopausal	360 (66.6)	242 (64.3)		26 (61.9)	54 (50.5)		26 (68.4)	83 (58.0)	
NA	8 (1.5)	4 (1.1)		1 (2.4)	–		–	1 (0.7)	
Tumor size (cm), *n* (%)									
<2	250 (46.4)	158 (42.1)	NS	14 (33.3)	29 (27.1)	NS	8 (21.1)	36 (25.2)	NS
2 to 5	261 (48.2)	198 (52.5)		23 (54.8)	67 (62.6)		27 (71.1)	89 (62.2)	
>5	22 (4.1)	14 (3.7)		4 (9.5)	10 (9.3)		1 (2.6)	13 (9.1)	
NA	7 (1.3)	6 (1.6)		1 (2.4)	1 (0.9)		2 (5.3)	5 (3.5)	
Tumor grade, *n* (%)									
1	144 (26.7)	39 (10.4)	<0.0001	2 (4.8)	2 (1.9)	0.0036	1 (2.6)	1 (0.7)	0.003
2	311 (57.5)	226 (60.0)		27 (64.3)	43 (40.2)		19 (50.0)	38 (26.6)	
3	47 (8.7)	91 (24.3)		10 (23.8)	55 (51.4)		13 (34.2)	90 (62.9)	
NA	38 (7.1)	20 (5.3)		3 (7.1)	7 (6.5)		5 (13.2)	14 (9.8)	
Lymph nodes, *n* (%)									
0	293 (54.2)	192 (50.9)	NS	15 (35.7)	48 (44.9)	NS	13 (34.2)	77 (53.8)	NS
1 to 3	145 (26.9)	109 (29.1)		16 (38.1)	28 (26.2)		13 (34.2)	36 (25.2)	
≥4	101 (18.7)	73 (19.5)		11 (26.2)	30 (28.0)		11 (28.9)	27 (18.9)	
NA	1 (0.2)	2 (0.5)		–	1 (0.9)		1 (2.6)	3 (2.1)	
Chemotherapy, *n* (%)									
No	275 (50.8)	174 (46.1)	NS	10 (23.8)	27 (25.2)	NS	9 (23.7)	20 (14.0)	NS
Yes	260 (48.2)	199 (53.1)		31 (73.8)	79 (73.8)		29 (76.3)	121 (84.6)	
NA	5 (0.9)	3 (0.8)		1 (2.4)	1 (0.9)		–	2 (1.4)	
Hormonotherapy, *n* (%)									
No	106 (19.5)	67 (17.6)	NS	22 (52.4)	45 (42.1)	NS	19 (50.0)	100 (69.9)	NS
Yes	432 (80.1)	307 (81.9)		20 (47.6)	62 (57.9)		19 (50.0)	43 (30.1)	
NA	2 (0.4)	2 (0.5)		–	–		–	–	
Local therapy, *n* (%)									
Mastectomy alone	172 (31.9)	105 (28.0)		19 (45.2)	30 (28.0)		20 (52.6)	36 (25.2)	
Mastectomy + RT	61 (11.3)	36 (9.6)	NS	7 (16.7)	16 (15.0)	NS	7 (18.4)	23 (16.1)	NS
Lumpectomy alone	28 (5.2)	13 (3.5)		1 (2.4)	8 (7.5)		–	5 (3.5)	
Lumpectomy + RT	279 (51.6)	222 (58.9)		15 (35.7)	53 (49.5)		11 (28.9)	79 (55.2)	

^a^HER2, human epidermal growth factor receptor 2; NA, not available; NS, not significant; RT, radiotherapy. ^b^*P *values were calculated using the χ^2 ^test. ^c^Two cases with Ki-67 were not available. ^d^One case with Ki-67 was not available.

**Table 3:** **Patient characteristics for breast cancer intrinsic subtypes defined by six biomarkers**^**a**^

**Characteristics**	**Luminal A and luminal B ** **(*N*** **= 916)**			**Luminal HER2 (*N*** **= 77)**			**HER2-enriched ** **(*N*** **= 74)**			**Basal-like ** **(*N*** **= 133)**			**TNP nonbasal** ** (*N*** **= 49)**		
	**Ki-67 <14% (*N*** **= 540)**	**Ki-67 ≥14% (*N*** **= 376)**	***P***^**b**^	**Ki-67 <14% (*N*** **= 18)**^**c**^	**Ki-67 ≥14% (*N*** **= 57)**^**c**^	***P***^**b**^	**Ki-67 <14% (*N*** **= 24)**	**Ki-67 ≥14% (*N*** **= 50)**	***P***^**b**^	**Ki-67 <14% (*N*** **= 13)**	**Ki-67 ≥14% (*N*** **= 120)**	***P***^**b**^	**Ki-67 <14% (*N*** **= 25)**^**d**^	**Ki-67 ≥14% (*N*** **= 23)**^**d**^	***P***^**b**^
Age (years), *n* (%)															
<40	33 (6.1)	32 (8.5)	NS	2 (11.1)	10 (17.5)	NS	2 (8.3)	5 (10.0)	NS	1 (7.7)	18 (15.0)	NS	4 (16.0)	4 (17.4)	NS
40 to 55	197 (36.5)	139 (37.1)		5 (27.8)	24 (42.1)		9 (37.5)	25 (50.0)		3 (23.1)	47 (39.2)		6 (24.0)	11 (47.8)	
>55	310 (57.3)	205 (54.4)		11 (61.1)	23 (40.4)		13 (54.2)	20 (40.0)		9 (69.2)	55 (45.8)		15 (60.0)	8 (34.8)	
Menopausal status, *n* (%)															
Premenopausal	172 (31.9)	130 (34.7)	NS	6 (33.3)	25 (43.9)	NS	9 (37.5)	28 (56.0)	NS	4 (30.8)	50 (41.7)	NS	8 (32.0)	9 (39.1)	NS
Postmenopausal	360 (66.6)	242 (64.3)		11 (61.1)	32 (56.1)		15 (62.5)	22 (44.0)		9 (69.2)	70 (58.3)		17 (68.0)	13 (56.5)	
NA	8 (1.5)	4 (1.1)		1 (5.6)	–		–	–		–	–		–	1 (4.3)	
Tumor size (cm), *n* (%)															
<2	250 (46.4)	158 (42.1)	NS	8 (44.2)	18 (31.6)	NS	6 (25.0)	11 (22.0)	NS	2 (15.4)	30 (25)	NS	6 (24.0)	6 (26.1)	NS
2 to 5	261 (48.2)	198 (52.5)		9 (50.0)	36 (63.2)		14 (58.3)	31 (62.0)		10 (76.9)	77 (64.2)		17 (68.0)	12 (52.2)	
>5	22 (4.1)	14 (3.7)		–	3 (5.3)		45 (16.7)	7 (14.0)		–	8 (6.7)		1 (4.0)	5 (21.7)	
NA	7 (1.3)	6 (1.6)		1 (5.6)	–		–	1 (2.0)		1 (7.7)	5 (4.2)		1(4.0)	–	
Tumor grade, *n* (%)															
1	144 (26.7)	39 (10.4)	<.0001	1 (5.9)	2 (3.8)	.004	1 (4.2)	–	NS	–	1 (0.8)	NS	1 (4.0)	–	.005
2	311 (57.5)	226 (60.0)		15 (88.2)	26 (50.0)		12 (50.0)	17 (34.0)		4 (30.8)	32 (26.7)		15 (60.0)	6 (26.1)	
3	47 (8.7)	91 (24.3)		1 (5.9)	24 (46.2)		9 (37.5)	31 (62.0)		8 (61.5)	75 (625)		5 (20.0)	15 (65.2)	
NA	38 (7.1)	20 (5.3)		–	–		2 (8.3)	2 (4.0)		1 (7.7)	12 (10.0)		4 (16.0)	2 (8.7)	
Lymph nodes, *n* (%)															
0	293 (54.2)	192 (50.9)	NS	9 (50.0)	31 (54.4)	NS	6 (25.0)	17 (34.0)	NS	3 (23.1)	64 (53.3)	NS	10 (40.0)	13 (56.5)	NS
1 to 3	145 (26.9)	109 (29.1)		7 (38.9)	18 (31.6)		9 (37.5)	10 (20.0)		5 (38.5)	32 (26.7)		8 (32.0)	4 (17.4)	
≥4	101 (18.7)	73 (19.5)		2 (11.1)	8 (14.0)		9 (37.5)	22 (44.0)		5 (38.5)	23 (19.2)		6 (24.0)	4 (17.4)	
NA	1 (0.2)	2 (0.5)		–	–		–	1 (2.0)		–	–		1 (4.0)	2 (8.7)	
Chemotherapy, *n* (%)															
No	275 (50.8)	174 (46.1)	NS	8 (44.4)	16 (28.1)	NS	2 (8.3)	11 (22.0)	NS	2 (15.4)	16 (13.3)	NS	7 (28.0)	7 (17.4)	NS
Yes	260 (48.2)	199 (53.1)		10 (55.6)	41 (71.9)		21 (87.5)	38 (76.0)		11 (84.6)	102 (85.0)		18 (72.0)	19 (82.6)	
NA	5 (0.9)	3 (0.8)		–	–		1 (4.2)	1 (2.0)		–	2 (1.7)		–	–	
Hormonotherapy, *n* (%)															
No	106 (19.5)	67 (17.6)	NS	4 (22.2)	13 (22.8)	NS	18 (75.0)	32 (64.0)	NS	9 (69.2)	86 (71.7)	NS	10 (40)	14 (60.9)	
Yes	432 (80.1)	307 (81.9)		14 (77.8)	44 (77.2)		6 (25.0)	18 (36.0)		4 (30.8)	34 (28.3)		15 (60)	9 (39.1)	
NA	2 (0.4)	2 (0.5)		–	–		–	–		–	–		–	–	
Local therapy, *n* (%)															
Mastectomy alone	172 (31.9)	105 (28.0)		7 (38.9)	14 (24.6)		12 (50.0)	16 (32.0)		5 (38.5)	28 (23.3)		15 (60.0)	8 (34.8)	
Mastectomy + RT	61 (11.3)	36 (9.6)	NS	1 (5.6)	5 (8.8)	NS	6 (25.0)	11 (22.0)	NS	3 (23.1)	17 (14.2)	NS	4 (16.0)	6 (26.1)	NS
Lumpectomy alone	28 (5.2)	13 (3.5)		1 (5.6)	4 (7.0)		–	4 (8.0)		–	5 (4.2)		–	–	
Lumpectomy + RT	279 (51.6)	222 (58.9)		9 (50.0)	34 (59.6)		6 (25.0)	19 (38.0)		5 (38.5)	70 (58.3)		6 (24.0)	9 (39.1)	

^a^HER2, human epidermal growth factor receptor 2; NA, not available; NS, not significant; RT, radiotherapy. ^b^*P *values were calculated using the χ^2 ^test. ^c^Two cases with Ki-67 NA. ^d^One case with Ki-67 NA.

Among patients with the various intrinsic subtypes, there were significant differences in histological grade depending on the proliferation rate defined by the Ki-67 index (Tables 2 and 3).

### Analysis of actuarial breast cancer–free survival by intrinsic subtype and proliferation rate

**Figure 2:**
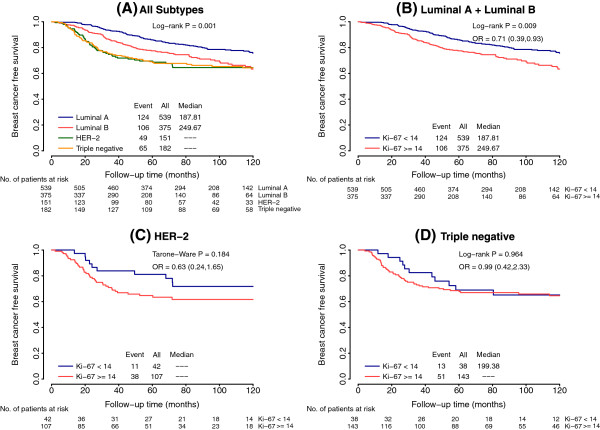
**Kaplan-Meier curves for breast cancer–free survival based on intrinsic subtypes (four markers) and proliferation rate. (A)** Whole series according to the four intrinsic subtypes. **(B)** Luminal tumors according to Ki-67 value. **(C)** HER2-enriched tumors according to Ki-67 value. **(D)** Triple negative tumors according to Ki-67 value. HER2, human epidermal growth factor receptor 2; OR, odds ratio.

**Figure 3:**
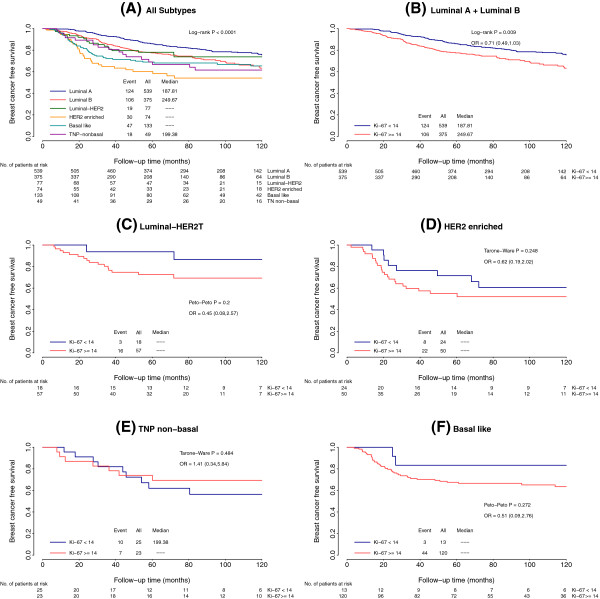
**Kaplan-Meier curves for breast cancer–free survival based on intrinsic subtypes (six markers) and proliferation rate. (A)** Whole series according to six intrinsic subtypes. TNP, triple-negative phenotype. **(B)** Luminal tumors according to Ki-67 value. OR, odds ratio. **(C)** Luminal human epidermal growth factor receptor 2 (HER2) tumors according to Ki-67 value. **(D)** HER2-enriched tumors according to Ki-67 value. **(E)** Triple-negative-nonbasal tumors according to Ki-67 value. **(F)** Basal-like tumors according to Ki-67 value.

The molecular subtypes differed significantly in BCFS when the four-biomarker definition was used (Figure 2A), with luminal A cases exhibiting the longest survival *(P =* 0.001). Analysis of BCFS in each intrinsic subtype according to Ki-67 value revealed a significant difference in the luminal subtypes (odds ratio (OR) = 0.71, 95% CI = 0.39 to 0.93; *P =* 0.009 (luminal A vs. luminal B)) (Figure 2B), but not in the HER2-enriched subtype (OR = 0.63, 95% CI = 0.24 to 1.65; *P* = 0.1) (Figure 2C) or the triple-negative subtype (OR = 0.96, 95% CI = 0.42 to 2.33; *P* = 0.9) (Figure 2D). Similar results were obtained when the six-immunomarker definition was used (Figure 3).

In Table 4, the cumulative 3-year and 5-year BCFS rates for the four intrinsic subtypes are listed. At 3 years, the absolute difference in BCFS between the luminal A and the luminal B phenotypes was 7.6%, which is comparable to the difference found at 5 years (8.7%). Similar data were found regarding the luminal A and the HER2-enriched phenotypes (19.5% and 17.1%, respectively) and the luminal A and triple-negative phenotypes (18.3% and 17.4%, respectively). The results exhibited similar trends when the six-marker definition was used (Table 5).

**Table 4:** **Estimated cumulative breast cancer–free survival (four-marker definition)**^**a**^

**Intrinsic subtype**	**2-year BCFS**	**95% CI**	**3-year BCFS**	**95% CI**	**5-year BCFS**	**95% CI**
Luminal A	96.7%	95.2% to 98.3%	92.8%	90.6% to 95.0%	85.8%	82.7% to 88.9%
Luminal B	91.5%	88.6% to 94.4%	85.2%	81.6% to 89.0%	77.1%	72.8% to 81.7%
HER2-enriched	81.2%	75.0% to 87.8%	73.3%	66.4% to 81.0%	68.7%	61.4% to 76.8%
Triple-negative	83.0%	77.7% to 88.8%	74.5%	68.3% to 81.2%	68.4%	61.8% to 75.7%

^a^BCFS, breast cancer–free survival; CI, confidence interval; HER2, human epidermal growth factor 2. All values are statistically significant.

**Table 5:** **Estimated cumulative breast cancer–free survival (six-marker definition)**^**a**^

**Intrinsic subtype**	**2-year BCFS**	**95% CI**	**3-year BCFS**	**95% CI**	**5-year BCFS**	**95% CI**
Luminal A	96.7%	95.2% to 98.3%	92.8%	90.6% to 95.0%	85.8%	82.7% to 88.9%
Luminal B	91.5%	88.6% to 94.4%	85.2%	81.6% to 89.0%	77.1%	72.8% to 81.7%
Luminal HER2	89.2%	82.5% to 96.6%	81.0%	72.4% to 90.5%	77.1%	68.8% to 72.4%
HER2-enriched	72.4%	62.5% to 83.8%	63.9%	53.5% to 76.3%	58.3%	47.6% to 71.5%
Basal-like	80.8%	74.3% to 87.9%	72.2%	64.9% to 80.4%	68.1%	60.5% to 76.7%
TNP nonbasal	89.3%	80.9% to 98.6%	80.6%	70.0% to 92.8%	66.7%	54.2% to 82.1%

^a^BCFS, breast cancer–free survival; CI, confidence interval; HER2, human epidermal growth factor 2; TNP, triple-negative phenotype. All values are statistically significant.

### Analysis of recurrence prognostic factors and variations in recurrence risk over time

**Figure 4:**
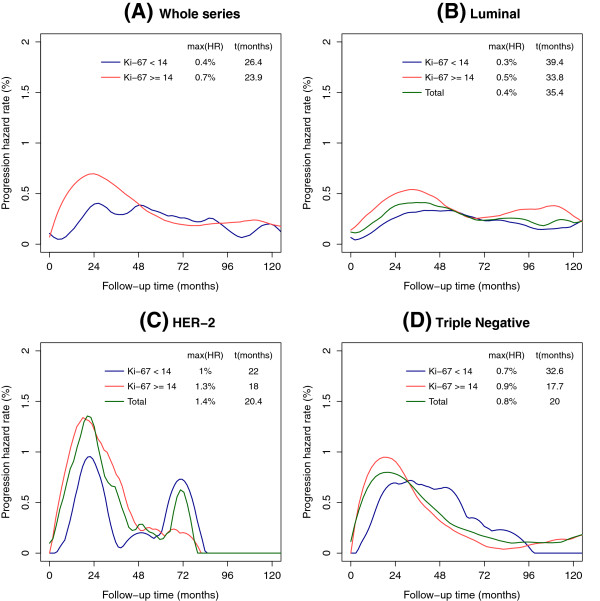
**Recurrence hazard rate functions for intrinsic subtypes (four markers) and proliferation rate. (A)** Whole series according to Ki-67 value. **(B)** Luminal tumors as a whole and according to Ki-67 value. **(C)** Human epidermal growth factor 2 (HER2)-enriched tumors as a whole and according to Ki-67 value. **(D)** Triple-negative tumors as a whole and according to Ki-67 value. max(HR), maximum hazard rate.

The final model for multivariate analyses of BCFS for patients classified using the four biomarkers revealed that tumor size, lymph node status, type of local therapy, use of adjuvant endocrine therapy and intrinsic subtypes were significant independent predictors of disease recurrence (Table 6). The analysis of compliance with proportional hazards assumptions were rejected (*P* < 0.0001), indicating that recurrence risks were not proportional over time. To further explore this issue, an analysis of the hazards function according to the intrinsic subtypes was performed, and the resulting smoothed curves are shown in Figure 4. We also investigated whether these patterns of relapse differed regarding the proliferation rate. The luminal A cases, with Ki-67 <14%, had a progressive increased risk that approached 0.3% (95% CI = 0.2% to 0.4%) at 39.4 months, with the curve remaining nearly steady during the rest of follow-up (Figure 4B). However, luminal B patients, with Ki-67 ≥14%, displayed a different pattern of recurrence. A maximum risk of 0.5% (95% CI = 0.4% to 0.6%) was reached at 33.8 months, and a second peak of late recurrence risk (0.3%; 95% CI = 0.2% to 0.5%) appeared at 112 months (Figure 4B). For the HER2-enriched subtype, the first peak occurred at approximately the same time, nearly 20 months for both groups, but the maximum risk was 1.0% (95% CI = 0.5% to 2%) in patients with Ki-67 <14% and a maximum risk of 1.3% (95% CI = 0.9% to 3.1%) in those cases with Ki-67 ≥14%. A second risk peak at 72 months appeared for both HER2 populations, but, interestingly, the magnitude in this case seems to be higher in the HER2-enriched group with Ki-67 <14% (0.75% risk; 95% CI = 0.3% to 1.8%) than with Ki-67 ≥14% (0.25% risk; 95% CI = 0.08% to 2.7%) (Figure 4C). Triple-negative cases with low Ki-67 exhibited a smooth curve. After a maximum risk of 0.7% (95% CI: 0.4% to 1.7%) reached at 32.6 months, the curve remained nearly steady until 52 months, at which point it started to decline. The maximum recurrence risk for triple-negative and high Ki-67 patients was reached at 17.7 months (0.9% risk; 95% CI = 0.6% to 1.1%), decreased to 0.3% by 48 months (95% CI = 0.2% to 0.5%) and was minimal at 80 months (0.04% risk; 95% CI = 0.007% to 0.2%) (Figure 4D).

**Table 6:** **Final multivariate Cox analysis of breast cancer–free survival with four-biomarker definition of intrinsic subtypes**^**a**^

**Variables**	**HR**	**95% CI**	***P***^**b**^
Tumor size, cm			
<2	1.0		
2 to 5	1.6	1.2 to 2.1	0.0004
>5	1.9	1.2 to 3.1	0.005
Lymph nodes			
0	1.0		
1 to 3	1.4	1.0 to 1.8	0.01
≥4	2.8	2.1 to 3.7	<0.0001
Local therapy			
Mastectomy alone	1.0		
Mastectomy + RT	1.1	0.7 to 1.5	0.6
Lumpectomy alone	1.2	0.6 to 2.1	0.5
Lumpectomy + RT	0.6	0.4 to 0.8	0.0005
Endocrine therapy			
Yes	1.0		
No	0.7	0.5 to 0.9	0.01
Subtype			
Luminal A	1.0		
Luminal B	1.3	1.0 to 1.7	0.02
HER2-enriched	1.4	1.0 to 2.0	0.04
Triple-negative	1.3	0.9 to 1.8	0.09

^a^CI, confidence interval; HER2, human epidermal growth factor receptor 2; HR, hazard ratio; RT, radiotherapy. ^b^All likelihood ratio, Wald and logrank statistical tests were two-sided.

The recurrence hazard rates for the intrinsic subtypes at different time points are listed in Table 7, showing their respective hazard ratios and considering luminal A as the reference value. It is noteworthy that the information is quite different from data derived from actuarial BCFS, in which the absolute differences between each intrinsic subtype and luminal A were comparable at two time points: 36 and 60 months. The initial risk for luminal B is about twice that for luminal A, and, after 36 months, this risk decreases slowly. After the eighth year, however, the recurrence risk for luminal B increases to more than twice that of luminal A. For HER2-enriched patients, the risk is nearly fivefold that of luminal A during the first 2 years and double at 36 months. Between the fourth and the fifth years after surgery, however, the recurrence risk for HER2-enriched patients is lower than that for luminal A patients. After that time, the recurrence risk for HER2-enriched patients increases, becoming more than twice that of luminal A. The risk of recurrence for triple-negative tumors is three times higher than that for luminal A during the first 2 years and nearly double at 36 months; however, at 60 months, the recurrence risk is similar between the two subtypes.

**Table 7:** **Intrinsic subtypes’ recurrence hazard rates over time and hazard ratios (Four markers definition)**^**a**^

**Years after surgery**	**Luminal A hazard rate**^**b**^	**HR**	**Luminal B hazard rate**	**HR**	**HER2-enriched hazard rate**	**HR**	**Triple-negative hazard rate**	**HR**
2	0.26 (0.2 to 0.3)	1	0.50 (0.4 to 0.6)	1.9	1.26 (0.8 to 2.0)	4.8	0.78 (0.6 to 1.0)	3.0
3	0.32 (0.2 to 0.4)	1	0.54 (0.4 to 0.6)	1.7	0.63 (0.3 to 1.3)	2.0	0.60 (0.4 to 0.8)	1.9
4	0.33 (0.2 to 0.4)	1	0.43 (0.3 to 0.5)	1.3	0.28 (0.1 to 0.9)	0.8	0.39 (0.2 to 0.5)	1.2
5	0.30 (0.2 to .04)	1	0.29 (0.2 to 0.4)	1.0	0.14 (.02 to 1.0)	0.4	0.24 (0.1 to 0.4)	0.8
6	0.23 (0.1 to 0.3)	1	0.26 (0.2 to 0.5)	1.1	0.62 (0.2 to 1.7)	2.6	0.17 (.06 to 0.3)	0.7
7	0.23 (0.1 to 0.3)	1	0.31 (0.2 to 0.6)	1.4	NA	NA	0.11 (.04 to 0.3)	0.5
8	0.17 (0.1 to 0.2)	1	0.35 (0.2 to 0.7)	2.0	NA	NA	0.11 (.04 to 0.3)	0.6
9	0.15 (0.1 to 0.3)	1	0.38 (0.2 to 0.7)	2.5	NA	NA	0.10 (.03 to 0.3)	0.7
10	0.19 (0.1 to 0.4)	1	0.28 (0.1 to 0.6)	1.5	NA	NA	0.16 (.06 to 0.4)	0.8

^a^HR, hazard ratio; HER2, human epidermal growth factor receptor 2; NA, not available. ^b^Reference value. Note: 95% confidence intervals are presented in parentheses.

**Figure 5:**
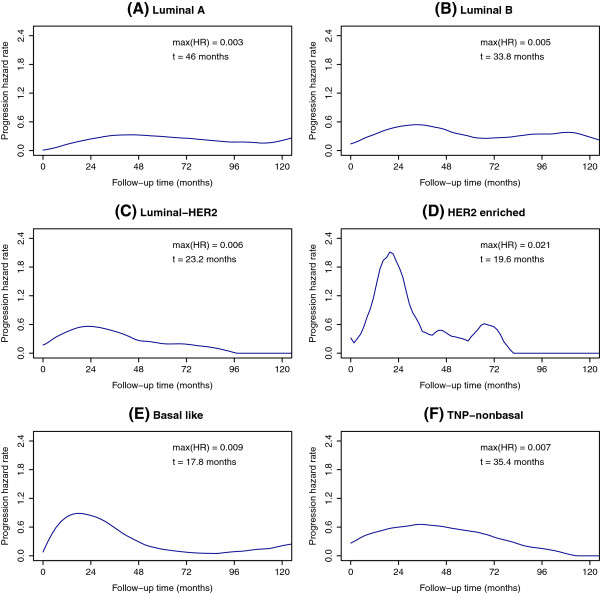
**Recurrence hazard rate functions for intrinsic subtypes (six markers). (A)** Luminal A tumors. **(B)** Luminal B tumors. **(C)** Luminal human epidermal growth factor receptor 2 (HER2) tumors. **(D)** HER2-enriched tumors. **(E)** Triple-negative phenotype (TNP) nonbasal tumors. **(F)** Basal-like tumors. max(HR), maximum hazard rate.

Similar results were obtained when multivariate analysis was performed with intrinsic subgroup populations defined by the six biomarkers (Table 8). In the same way, each intrinsic subtype displayed a specific pattern of recurrence over time, although the analysis based on the Ki-67 value was not performed, owing to the size of the subgroups, which were too small to make estimates realistic (Figure 5 and Table 9).

**Table 8:** **Final multivariate Cox analysis of breast cancer–free survival with six-biomarker definition of intrinsic subtypes**^**a**^

**Variable**	**HR**	**95% CI**	***P***^**b**^
Tumor size, cm			
<2	1.0		
2 to 5	1.6	1.2 to 2.1	0.0003
>5	2.0	1.2 to 3.2	0.005
Lymph nodes			
0	1.0		
1 to 3	1.4	1.0 to 1.8	0.01
≥4	2.8	2.1 to 3.7	<0.0001
Local therapy			
Mastectomy alone	1.0		
Mastectomy + RT	1.1	0.8 to 1.5	0.6
Lumpectomy alone	1.2	0.7 to 2.1	0.5
Lumpectomy + RT	0.6	0.5 to 0.8	0.0009
Hormonotherapy			
Yes	1.0		
No	0.7	0.5 to 0.9	0.01
Subtype			
Luminal A	1.0		
Luminal B	1.4	1.1 to 1.7	0.02
Luminal HER2	1.4	0.8 to 2.2	0.2
HER2-enriched	1.5	0.9 to 2.2	0.08
Basal-like	1.2	0.8 to 1.8	0.3
TNP nonbasal	1.6	0.9 to 2.6	0.06

^a^CI, confidence interval; HR, hazard ratio; HER2, human epidermal growth factor receptor 2; RT, radiotherapy; TNP, triple-negative phenotype. ^b^All likelihood ratio, Wald and logrank statistical tests were two-sided.

**Table 9:** **Intrinsic subtype recurrence hazard rates over time and hazard ratios (six-marker definition)**^**a**^

**Years after surgery**	**Luminal A hazard rate**^**b**^	**HR**	**Luminal B hazard rate**	**HR**	**Luminal HER2 hazard rate**	**HR**	**HER2-enriched hazard rate**	**HR**	**Basal-like hazard rate**	**HR**	**TNP nonbasal hazard rate**	**HR**
2	0.24 (0.2 to 0.3)	1	0.50 (0.4 to 0.6)	2.05	0.56 (0.3 to 0.9)	2.30	1.83 (1.1 to 3.1)	7.53	0.84 (0.6 to 1.2)	3.46	0.60 (0.3 to 1.1)	2.46
3	0.32 (0.2 to 0.4)	1	0.54 (0.4 to 0.6)	1.68	0.46 (0.2 to 0.8)	1.43	0.45 (0.1 to 1.4)	1.41	0.58 (0.4 to 0.8)	1.83	0.66 (0.4 to 1.1)	2.06
4	0.33 (0.2 to 0.4)	1	0.43 (0.3 to 0.5)	1.32	0.27 (0.1 to 0.5)	0.82	0.41 (0.1 to 1.6)	1.27	0.29 (0.2 to 0.5)	0.90	0.60 (0.3 to 1.1)	1.84
5	0.29 (0.2 to 0.3)	1	0.29 (0.2 to 0.4)	1.00	0.20 (0.07 to 0.5)	0.68	0.32 (0.06 to 1.6)	1.09	0.14 (0.05 to 0.3)	0.47	0.51 (0.3 to 1.0)	1.75
6	0.26 (0.1 to 0.3)	1	0.26 (0.1 to 0.4)	1.02	0.19 (0.05 to 0.6)	0.72	0.55 (0.1 to 2.2)	2.12	0.08 (0.03 to 0.2)	0.29	0.39 (0.2 to 0.9)	1.51
7	0.21 (0.1 to 0.3)	1	0.31 (0.2 to 0.5)	1.43	0.13 (0.03 to 0.5)	0.59	NA	NA	0.05 (0.01 to 0.3)	0.24	0.25 (0.08 to 0.8)	1.16
8	0.18 (0.1 to 0.2)	1	0.35 (0.2 to 0.6)	1.95	0.01 (0.00 to 0.04)	0.06	NA	NA	0.09 (0.02 to 0.3)	0.49	0.15 (0.04 to 0.6)	0.86
9	0.16 (0.1 to 0.3)	1	0.38 (0.2 to 0.7)	2.33	NA	NA	NA	NA	0.14 (0.04 to 0.4)	0.84	0.05 (0.01 to 0.2)	0.30
10	0.21 (0.1 to 0.3)	1	0.28 (0.1 to 0.6)	1.33	NA	NA	NA	NA	0.21 (0.08 to 0.6)	1.00	NA	NA

^a^HER2, human epidermal growth factor receptor 2; HR, hazard ratio; NA, not available; TNP, triple-negative phenotype. ^b^Reference value. Note: 95% confidence interval is represented between the parentheses.

## Discussion

Our work highlights the importance of the proliferation pathway in the prognosis of early breast cancer intrinsic subtypes through the analysis of patient outcomes in different and complementary ways.

On the basis of genomics-defined luminal tumors, it has been possible to establish a value of Ki-67 with prognostic utility usefulness in distinguishing luminal tumors A and B [12]. Moreover, several studies have confirmed the prognostic usefulness of these intrinsic subtypes defined by four immunohistochemical markers [6-10] or six immunohistochemical markers [8,13-15]. However, the value of Ki-67 as a prognostic marker in the other intrinsic subtypes, such as triple-negative and HER2-enriched, is not clear. In our study, only the luminal population demonstrated significant differences in actuarial BCFS according to Ki-67 value. No significant differences were found in triple-negative and HER2-enriched tumors. It is possible that these findings are based on our use of a Ki-67 cutoff obtained in luminal tumors. Nevertheless, Aleskandarany *et al*. also failed to detect a significant difference in actuarial survival, despite using different cutoffs defined specifically for both triple-negative and HER2-enriched tumors [31].

To improve the results obtained using adjuvant therapy in breast cancer, it is important to develop methods for accurately determining which patients need some kind of treatment. Furthermore, it is necessary to establish the best treatment option for each patient and the best timing of treatment administration, because previous studies have shown that there are significant differences in the timing of disease recurrence, depending on tumor characteristics. Using a cumulative survival methodology, researchers in various studies have found that, in triple-negative and HER2 phenotypes, most relapses occur during the first 3 years of follow-up, whereas in the luminal subtypes, a significant number of recurrences occur in subsequent years [32-38]. Nevertheless, the use of the hazard function analysis methodology defines, in much greater detail, the changes in the risk of relapse over time, highlighting when a recurrence occurs rather than simply calculating the overall recurrence risk. In our Kaplan-Meier data, there were no appreciable absolute differences in 3-year and 5-year BCFS between luminal A and luminal B cases (7.6% and 8.7%, respectively), luminal A and HER2-enriched cases (19.5% and 17.1%, respectively) or luminal A and triple-negative cases (18.3% and 17.4%, respectively). However, the hazard function analysis detected noteworthy differences in the relapse risk between these subgroups at the same time points. Luminal B patients had about twice the recurrence risk of luminal A patients 3 years after surgery, whereas the hazard ratio was only 1.0 at 5 years. In HER2-enriched tumors, the recurrence hazard ratio in comparison with luminal A was 2.0 at 3 years and 0.4 at 5 years postsurgery. Also, triple-negative patients had different recurrence risks when luminal A data were considered as reference values (1.9 at 3 years and 0.8 at 5 years).

A visual inspection of the recurrence hazard curves presented herein shows that each intrinsic subtype has a particular pattern of relapse over time. More importantly, these patterns change depending on the level of activation of the proliferation pathway as determined by Ki-67. To date, few studies have examined the temporal pattern of recurrence in the various intrinsic subtypes, and the results of these studies have demonstrated hazard function curves similar to ours. Greater short-term risk between 1 and 3 years after surgery was observed in the triple-negative tumors [34,38-42], HER2-enriched tumors [38,40,42] and luminal B tumors [40,42], whereas the long-term risk was greater in the luminal subtypes [38,40,42]. Interestingly, a second peak of late recurrences was also observed in the HER2 phenotypes [38,42]. No data regarding differences that depend on the Ki-67 value are available, except those reported by Keam *et al*. [41]. Those authors analyzed a series of 109 triple-negative patients and used a Ki-67 cutoff of 10. Similar to our results, the low Ki-67 group showed a steady pattern and the high Ki-67 group displayed a sharp recurrence peak at 12 months.

The first peak of early relapses has been associated with surgery because the removal of the primary tumor could trigger the growth of clinically unapparent dormant micrometastatic foci [43]. Surgery could promote the growth of micrometastatic disease through several processes, such as an alteration in the angiogenic balance [43,44], surgical stress-induced neuroendocrine activation [45] or alteration of the immune response [46,47]. These mechanisms could influence particularly the disease course in intrinsic subtypes with high expression of proliferation pathways, such as HER2 or basal-like tumors. An increase of proliferation has been reported in HER2-positive patients with positive tumor margins after conservative surgery between the first and second tumor samples, as determined using Ki-67 immunohistochemistry, but this was not the case with HER2-negative cases [48].

The information provided by the study of the patterns of recurrence in early breast cancer would benefit patients in different ways. In this regard, our results could generate several hypotheses that, if confirmed in prospective randomized trials, would have noteworthy practical value. First of all, the surveillance after initial treatment could be fit to the expected recurrence pattern based on each intrinsic subtype. More important, however, is that the adjuvant treatment could be tailored more accurately according to each intrinsic subtype. Patients with tumors with high proliferation rates, such as HER2-enriched or basal-like, would benefit from more aggressive chemotherapy schedules (for example, dose-dense). Such types of chemotherapy could avoid some of the recurrences that appear during the first peak. Also, in these cases with high expression of proliferation pathways, treatment with novel inhibitors of the cell cycle (for example, palbociclib) could be especially useful. In addition, those patients with luminal HER2 subtype could benefit from a second treatment with trastuzumab to decrease the second peak of recurrence.

The essential strengths of our study are the detailed and careful analysis of BCFS data, which describes a specific relapse pattern for every intrinsic subtype as a whole and is distinguished by the level of proliferation pathway activation in a homogeneously managed series of patients representing a full spectrum of breast cancers, which is not always available in clinical trial–based samples. The main limitation of our study is the lack of availability of tumor samples from all patients. It could be argued that the use of different schemes of adjuvant chemotherapy could have caused less consistency in our results. Evidence from the studies that initially described the special recurrence pattern of early breast cancer suggest that the structure of this pattern is the same, regardless of the type of adjuvant therapy used [16-18]. In this regard, the only change we observed was the height of the recurrence peaks, but not their number or their shape. Data from a patient series in Milan, Italy [17,18], and from Eastern Cooperative Oncology Group coordinated studies [16], including patients treated only with surgery and patients treated with surgery plus several schemes of adjuvant chemotherapy, reproduced this recurrence structure with robustness.

The proliferation pathway plays a key role in the development of early recurrence after surgery in breast cancer, regardless of the intrinsic subtype involved. This conclusion is reinforced by the fact that our data were obtained by following a different statistical approach to survival analyses. Our results need to be corroborated in larger series of patients treated with current adjuvant systemic therapies; however, transferring knowledge regarding temporary patterns of recurrence in the development and design of future clinical trials in the adjuvant setting could be considered in establishing the timing or schedule of treatment administration that would be more effective. In addition, our data could have some impact on recommendations concerning patient follow-up.

## Conclusions

Breast cancer intrinsic subtypes using both four- and six-marker immunohistochemical panels and proliferation assessed by using Ki-67 were determined in a large and homogeneous cohort of patients collected prospectively. Our most important findings are that each intrinsic subtype displayed a specific pattern of recurrence and that the proliferation pathway played a key role in the development of early recurrence. These results point directly to adjuvant treatment approaches and clinical follow-up schedules for surveillance, suggesting that both should be different, depending on intrinsic subtype. Moreover, understanding of these distinct clinical patterns of relapse may lead to new biological insights into the development and management of breast cancer.

## Abbreviations

BCFS: Breast cancer–free survival; CISH: Chromogenic *in situ* hybridization; CK: Cytokeratin; EGFR: Epidermal growth factor receptor; ER: Estrogen receptor; HER2: Human epidermal growth factor receptor 2; PR: Progesterone receptor; TNP nonbasal: Triple-negative nonbasal phenotype.

## Competing interests

The authors declare that they have no competing interests.

## Authors’ contributions

EA, NR and JMJ contributed to the conception and design of the study. LPV, LV, VL, EG and MA contributed to the tissue microarray construction and immunohistochemical scoring. BP, BJ, NR, AM, ASM and LPR contributed to the acquisition and assembly of clinical data. EA, NR, JMJ and LF contributed to the analysis and interpretation of the data. NR, JMJ and EA contributed to the drafting of the manuscript. All authors approved the final manuscript.
